# The association between *HHEX* single-nucleotide polymorphism rs5015480 and gestational diabetes mellitus: A meta-analysis

**DOI:** 10.1097/MD.0000000000019478

**Published:** 2020-03-20

**Authors:** Xingjie Wang, Yuanlin Ding, Xinshan Zhang, Jiawei Rao, Haibin Yu, Haiyan Pan

**Affiliations:** School of Public Health, Guangdong Medical University, Dongguan, Guangdong Province, China.

**Keywords:** diabetes, gestational, meta-analysis, polymorphism, single nucleotide

## Abstract

**Objective:**

To evaluate the association between the rs5015480 single-nucleotide polymorphism of hematopoietically expressed homeobox (*HHEX*) and gestational diabetes mellitus (GDM) via meta-analysis.

**Methods:**

A comprehensive electronic search was performed of the PubMed, Springer, Science Direct, China National Knowledge Infrastructure (CNKI), Wanfang, and VIP databases for studies worldwide on the relationship between *HHEX* rs5015480 and GDM published up to July 2019. Rigorous inclusion and exclusion criteria were developed, and the quality of studies was assessed using the Newcastle–Ottawa scale, followed by heterogeneity evaluation using the *Q* test and *I*^2^ statistic and data pooling. A meta-analysis was then performed on the included studies using RevMan 5.3.

**Results:**

A total of 4 eligible case–control studies were included, involving a total of 1651 patients and 3513 controls. The meta-analysis showed the following odds ratios: C allele vs T allele, 1.24 (95% confidence interval [CI]: 1.12–1.38); CC genotype vs TT genotype, 1.65 (95% CI: 1.26–2.17); CC genotype vs CT genotype, 1.22 (95% CI: 1.00–1.50); and CC genotype vs CT + TT genotype, 1.32 (95% CI: 1.09–1.61).

**Conclusions:**

*HHEX* rs5015480 represents a risk factor for the development of GDM, and pregnant women carrying the CC genotype have an increased risk of GDM.

## 1 Introduction

Gestational diabetes mellitus (GDM) is one of the most common perinatal complications. The etiology of GDM is multifactorial, with many factors such as maternal health status and social psychology involved in its development and outcome.^[1,2]^ With the full implementation of China's two-child policy, the number of high-risk pregnant women has increased, and the incidence of GDM has continued to rise. The 2017 International Diabetes Federation (IDF) Diabetes Atlas showed that the global incidence of GDM was as high as 14.0%.^[3]^ The incidence of GDM in China has reached 18.9%. There is evidence that women with a history of GDM have an approximately 48% chance of developing diabetes in the next pregnancy^[4]^; 25% to 70% of women with GDM may develop true diabetes within 20 years and tend to experience serious consequences, including miscarriage and fetal growth restriction, and the newborns have significantly increased risk of developing obesity and diabetes in adulthood.^[5]^

In recent years, with the completion of the Human Genome Project, new breakthroughs have been made in molecular genetics research on GDM. Studies on the association between single-nucleotide polymorphisms (SNPs) of the hematopoietically expressed homeobox (*HHEX*) gene and the risk of GDM have been reported. Such studies have focused on the *HHEX* SNP rs5015480 but less on rs1111875; accordingly, rs5015480 was selected as the target in the present study.

Here, quantitative synthesis and comprehensive assessment of epidemiological studies on *HHEX* rs5015480 and GDM risk were performed using meta-analysis to provide a more objective basis for evidence-based medicine for investigating GDM etiology.

## 2 Materials and methods

### 2.1 Literature search

An electronic search was performed for articles written in English in the PubMed, Springer, and Science Direct databases using the relevant terms (“gestational diabetes mellitus" OR “GDM") AND (“Homeobox gene expression in hematopoietic stem cells" OR “HHEX") AND (“rs5015480" OR “single nucleotide polymorphism"). An electronic search was also performed for articles written in Chinese in the China National Knowledge Infrastructure (CNKI), Wanfang, and VIP databases using the relevant terms (“gestational diabetes mellitus" OR “GDM") AND (“Homeobox gene expression in hematopoietic stem cells" OR “HHEX") AND (“rs5015480" OR “single nucleotide polymorphism"). Epidemiological studies on the association between the *HHEX* SNP and GDM published worldwide up to July 2019 were retrieved. The included studies were collated according to first author, publication date, and regional characteristics; and we extracted information on genotype and allele distribution in cases and controls.

### 2.2 Inclusion and exclusion criteria

#### 2.2.1 Inclusion criteria

The inclusion criteria were as follows: epidemiological studies on the association between *HHEX* rs5015480 and GDM; well-designed cohort studies, case–control studies, case–cohort studies, and cross-sectional studies; sufficient genotype or allele frequency data were provided; all patients included were confirmed with GDM according to the criteria in International Association of Diabetes in Pregnancy Study Groups (IADPSG) and Obstetrics and Gynecology (8th edition); and the allele frequencies were in Hardy–Weinberg (H–W) equilibrium.

#### 2.2.2 Exclusion criteria

The exclusion criteria were as follows: abstracts, reviews, lectures, commentaries, and dissertations with duplicate publication; and studies with genotype or allele frequency data that were incomplete or that could not be extracted.

### 2.3 Data extraction

Two reviewers independently screened the studies according to the inclusion criteria. Disagreement was settled by discussion or by a third investigator. The following information was extracted from eligible studies: first author, publication date, definition and characteristics of subjects in case and control groups, total number of cases included, distribution and frequency of alleles and genotypes in cases and controls, and sources of genotyping samples.

### 2.4 Statistics

The methodological quality of the studies was evaluated using the Newcastle–Ottawa scale (NOS); all studies scoring >5 were included in the meta-analysis. RevMan 5.3 was used for heterogeneity testing (*Q* test and *I*^2^ statistic), forest plot generation, and publication bias analysis. Differences were considered statistically significant at *P* < .05.

## 3 Results

### 3.1 Basic characteristics of the included studies

**Figure 1:**
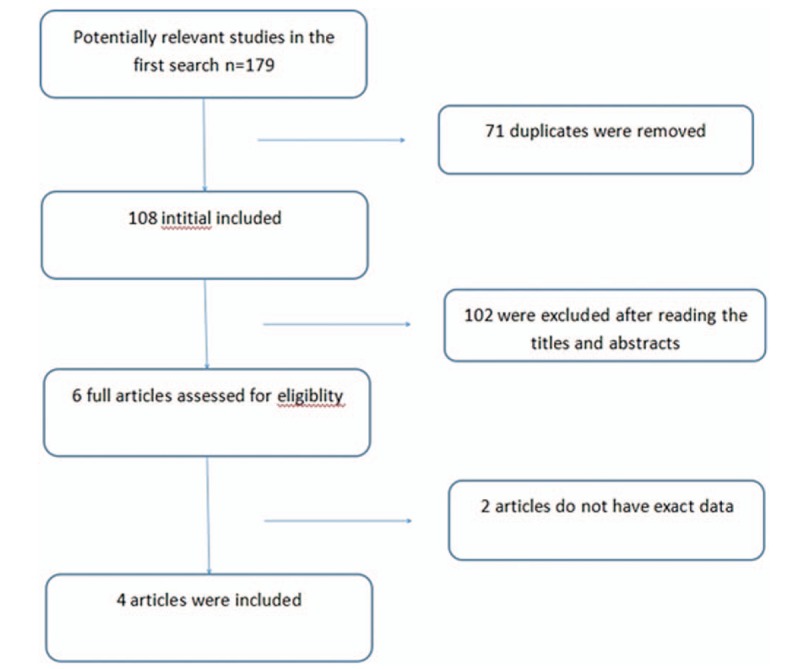
Flow diagram of study selection.

The 179 articles retrieved using the search terms were screened, and finally, a total of 4 case–control studies that met the inclusion criteria were included (Fig. 1). Of these, 1 was written in Chinese and 3 in English, and the studies involved 1651 patients and 3513 controls. The genotype distribution in all included studies was in H–W equilibrium, and the NOS scores were >5. Table 1 shows the basic characteristics of the included studies.

**Table 1:**
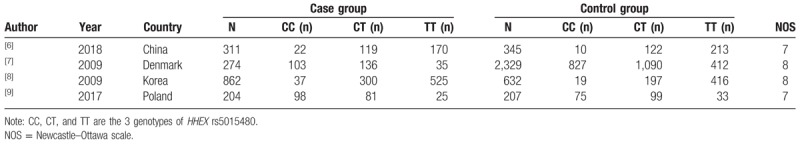
Basic characteristics of the included studies.

### 3.2 Results of meta-analysis

#### 3.2.1 Meta-analysis of C allele vs T allele of HHEX rs5015480

**Figure 2:**
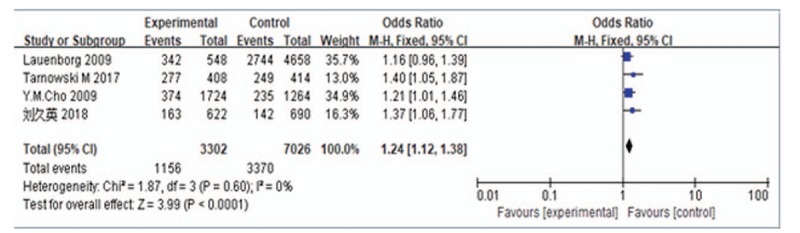
Forest plot of C allele vs T allele of *HHEX* rs5015480. CI = confidence intervals, HHEX = Homeobox gene expression in hematopoietic stem cells.

A meta-analysis was performed for the C vs T alleles (analysis was performed for all C and T alleles included in the studies). Assessment of the heterogeneity of the included studies showed Chi-square = 1.87, *P* = .60, and *I*^2^ = 0%, suggesting no heterogeneity in the allele distribution of the included studies; therefore, a fixed-effects model was used for data pooling. The results showed an odds ratio (OR) of 1.24 (95% confidence interval [CI]: 1.12–1.38), suggesting that *HHEX* rs5015480 is a risk factor for GDM development (Fig. 2).

#### 3.2.2 Meta-analysis of CC genotype vs TT genotype of HHEX rs5015480

**Figure 3:**
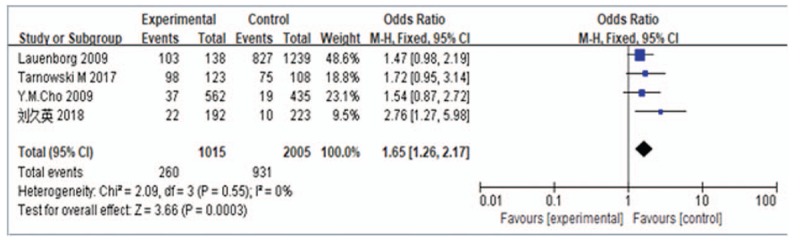
Forest plot of CC genotype vs TT genotype of *HHEX* rs5015480. CI = confidence intervals, HHEX = Homeobox gene expression in hematopoietic stem cells.

A meta-analysis was performed for the CC vs TT genotypes. Evaluation of the heterogeneity of the included studies showed Chi-square = 2.09, *P* = .55, and *I*^2^ = 0%, suggesting no heterogeneity in the genotype distribution of the included studies; therefore, a fixed-effects model was used for data pooling. The results showed an OR of 1.65 (95% CI: 1.26–2.17) (Fig. 3).

#### 3.2.3 Meta-analysis of CC phenotype vs CT phenotype of HHEX rs5015480

**Figure 4:**
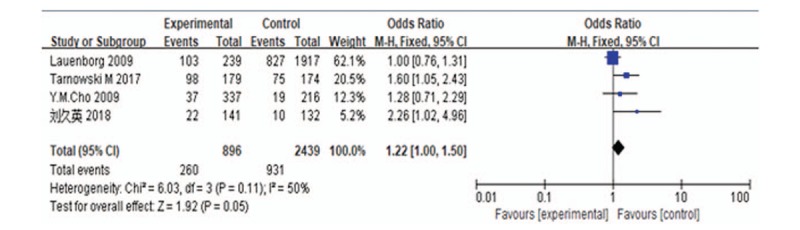
Forest plot of CC genotype vs TC genotype of *HHEX* rs5015480. CI = confidence intervals, HHEX = Homeobox gene expression in hematopoietic stem cells.

A meta-analysis was performed for the CC vs CT genotypes. Assessment of the heterogeneity of the included studies showed Chi-square = 6.03, *P* = .11, and *I*^2^ = 50%. The *P* value was >.1 and *I*^2^ = 50%, indicating no heterogeneity in the genotype distribution of the included studies; therefore, a fixed-effects model was used for data pooling. The results showed an OR of 1.22 (95% CI: 1.00–1.50) (Fig. 4).

#### 3.2.4 Meta-analysis of CC genotype vs TC + TT genotype of HHEX rs5015480

**Figure 5:**
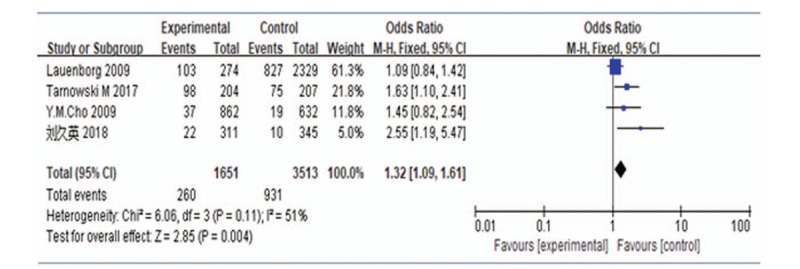
Forest plot of CC genotype vs TC + TT genotype of *HHEX* rs5015480. CI = confidence intervals, HHEX = Homeobox gene expression in hematopoietic stem cells.

A meta-analysis was performed for the CC vs CT + TT genotypes. Assessment of the heterogeneity of the included studies showed Chi-square = 6.06, *P* = .11, and *I*^2^ = 51%. The *P* value was >.1 and *I*^2^ was approximately 50%, indicating no heterogeneity in the genotype distribution of the included studies; therefore, a fixed-effects model was used for data pooling. The results showed an OR of 1.32 (95% CI: 1.09–1.61), suggesting increased risk of GDM development in pregnant women with the CC genotype (Fig. 5).

### 3.3 Sensitivity analysis

Sensitivity analysis was performed by iterative removal of each study and repeating the analysis. The results showed no significant changes in the pooled effects, indicating that the meta-analysis results were stable and reliable.

### 3.4 Publication bias

Due to the small number of articles included, publication bias was not assessed.

## 4 Discussion

The *HHEX* gene has been mapped to the 23.33 region in the long arm of chromosome 10 (10q23.33), with a total length of 5.7 kb and 4 introns.^[10]^ In recent years, several studies have confirmed that *HHEX* is a GDM susceptibility gene.^[11–14]^ Changes in *HHEX* expression, associated with its polymorphism, may contribute to altered paracrine regulation of insulin secretion. This causes reduced insulin secretion by β-cells, which in turn leads to diabetes. In addition, the C allele of *HHEX* rs5015480 has been linked to decreased pancreatic β-cell function, β-cell glucose sensitivity, and insulin secretion, as evaluated using a β-cell (homeostasis model assessment-B]) homeostasis model.^[15]^

Human genome-wide association studies have demonstrated that *HHEX* SNPs are associated with the risk for type 2 diabetes mellitus, which has been confirmed in a replication study in a different population.^[16]^ Researchers worldwide have started to study their association with GDM. However, the results have been inconsistent due to the differences in research methods or inclusion criteria, too small sample size, or great variation in sample size across the studies. Therefore, we performed a meta-analysis to increase the sample size and resolve the inconsistency between the results of individual studies and thereby obtain comprehensive assessment results closer to reality.

Here, a total of 4 eligible case–control studies were included, consisting of 1 Chinese article and 3 English articles and involving a total of 1651 patients and 3513 controls. All the subjects in the studies did not deviate from H–W equilibrium, suggesting good representativeness of subjects. In addition, the NOS scores indicated that the included studies had high methodological quality. The meta-analysis showed that the OR were as follows: C allele vs T allele, 1.24 (95% CI: 1.12–1.38); CC genotype vs TT genotype, 1.65 (95% CI: 1.26–2.17); CC genotype vs CT genotype, 1.22 (95% CI: 1.00–1.50); and CC genotype vs CT + TT genotype, 1.32 (95% CI: 1.09–1.61). The data show that *HHEX* rs5015480 is a risk factor for GDM, and pregnant women carrying the CC genotype have elevated risk of developing GDM. However, given the small number of relevant studies and populations involved, the generalization of this conclusion awaits additional studies.

The related studies have mainly focused on *HHEX* rs5015480 and rs1111875, and all studies on rs5015480 suggest that it is a risk factor for the development of GDM. For example, in a Korean population,^[8]^ the C allele of *HHEX* rs5015480 was associated with increased risk of GDM and decreased insulin secretion upon glucose challenge. In a Danish population,^[7]^*HHEX* rs1111875 was also associated with GDM. A study in Poland^[9]^ indicated that the C allele of *HHEX* rs5015480 might be a risk allele for GDM and was associated with increased body mass index (BMI) during pregnancy. However, there have been inconsistent conclusions on rs1111875 in China and other countries. In Korean population and Jining population of Shandong Province studied by Cho et al^[8]^ and He et al,^[17]^ respectively, the authors drew the same conclusion: the 3 genotypes (GG, GA, and AA) of *HHEX* rs1111875 were associated with GDM development. In contrast, Hu et al^[18]^ studied the population in Jiangsu Province and Liu et al^[6]^ studied the population in central China, and concluded that there was no association between rs1111875 and GDM susceptibility. The inconsistency regarding rs1111875 may be attributed to regional differences and small sample size. In the future, the sample size and multicenter cooperation in different regions should be increased to investigate the relationship between this locus and GDM susceptibility.

## Acknowledgments

We thank all the participants for their contribution to this work.

## Author contributions

**Conceptualization:** Xingjie Wang, Yuanlin Ding.

**Investigation:** Jiawei Rao, Xinshan Zhang.

**Supervision:** Haiyan Pan, Haibin Yu.

**Writing – original draft:** Xingjie Wang, Yuanlin Ding.

**Writing – review & editing:** Haiyan Pan, Haibin Yu.
